# Complete Genome Sequences of *Campylobacter jejuni* Strains Isolated from Retail Chicken and Chicken Gizzards

**DOI:** 10.1128/genomeA.01351-17

**Published:** 2017-11-22

**Authors:** Daya Marasini, Mohamed K. Fakhr

**Affiliations:** Department of Biological Science, The University of Tulsa, Tulsa, Oklahoma, USA

## Abstract

Genome sequences of *Campylobacter jejuni* FJ3124 and ZP3204 isolated from retail chicken gizzards and *Campylobacter jejuni* TS1218 isolated from retail chicken showed the presence of 1,694,324-, 1,763,161-, and 1,762,596-bp circular chromosomes, respectively. *Campylobacter jejuni* ZP3204 and TS1218 harbored large tetracycline resistance plasmids with type IV secretion systems.

## GENOME ANNOUNCEMENT

*Campylobacter* spp. have been reported to be prevalent in retail chicken livers and gizzards; however, the prevalence of these foodborne pathogens in chicken livers was higher than that in chicken gizzards (1). The majority of retail chicken samples are known to be contaminated with *Campylobacter jejuni* (2, 3). Recent advances in DNA sequencing technology have helped to increase understanding of the genomic structure of these retail isolates. Our laboratory was recently involved in the detection, molecular characterization, plasmid profiling, and whole-genome sequencing of *Campylobacter* spp. isolated from various retail meats (1, 3–10).

To our knowledge, only one *C. jejuni* genome strain, WP2202, of a chicken gizzard origin has been sequenced and reported (10). Hence, here we announce the complete genome sequences of two *Campylobacter jejuni* isolates from chicken gizzards and one from retail chicken. The three strains were previously isolated from retail poultry (1, 3). Total genomic DNA was extracted using the DNeasy blood and tissue kit (Qiagen, Inc., Valencia, CA) from a 72-h liquid culture of each of the *Campylobacter jejuni* strains sequenced in this study. A Nextera XT library preparation kit (Illumina, Inc., San Diego, CA) was used to prepare the library according to the manufacturer’s protocol. It was then run on a MiSeq desktop sequencer using an Illumina V2 reagent kit (Illumina). Coverage of 200× was obtained from the paired-end reads. CLC Genomics Workbench version 7.5.1 (Qiagen) was applied for raw data assembly, and genome alignment was performed using the Microbial Genome Finishing Module (Qiagen).

The complete genome sequences of the chicken gizzard isolates *Campylobacter jejuni* FJ3124 and ZP3204 revealed the presence of circular chromosomes of 1,694,324 and 1,763,161 bp, respectively. *Campylobacter jejuni* FJ3124 contained a total of 1,784 genes, 1,733 coding sequences (CDS), and 1,662 coding genes along with 56 RNAs and 71 pseudogenes. *Campylobacter jejuni* ZP3204 contained 1,906 genes, 1,852 CDS, and 1,764 coding genes with 54 RNAs and 88 pseudogenes. On the other hand, the chicken isolate *Campylobacter jejuni* TS1218 contained a circular chromosome of 1,762,596 bp and has 1,896 genes, 1,840 CDS, and 1,757 coding genes with 56 RNAs and 63 pseudogenes.

*Campylobacter jejuni* ZP3204 also contained two circular plasmids, pCJDM204L and PCJDM204S, of 44,436 and 5,257 bp, respectively. The larger plasmid contained a type IV secretion system and a tetracycline resistance gene with 47 CDS and 2 pseudogenes. The smaller plasmid contained 6 CDS with some hypothetical and replication proteins. *Campylobacter jejuni* TS1218 also contained one tetracycline resistance plasmid (43,077 bp) with a type IV secretion system and had 48 CDS and 2 pseudogenes.

It is noteworthy that the chromosomes of the chicken gizzard isolate *Campylobacter jejuni* ZP3204 and the chicken isolate *Campylobacter jejuni* TS1218 contain the genes coding for the type VI secretion system, which are homologous to previously reported plasmid-borne type VI secretion genes (10).

### Accession number(s).

The genome sequences of the chromosomes and plasmids for the 3 strains are available in GenBank with accession numbers as follows: chromosomes, CP017856 (*Campylobacter jejuni* ZP3204), CP017862 (*Campylobacter jejuni* FJ3124), and CP017860 (*Campylobacter jejuni* TS1218); plasmids, CP017854 (*Campylobacter jejuni* ZP3204 pCJDM204L), CP017855 (*Campylobacter jejuni* ZP3204 pCJDM204S), and CP017861 (*Campylobacter jejuni* TS1218 pCJDM218).
